# Sensory Processing as a Predictor of Leisure Participation in Early Adolescents

**DOI:** 10.3390/children8111005

**Published:** 2021-11-03

**Authors:** Young-Eun Choi, Hyerim Jung

**Affiliations:** 1 Department of Physical Therapy, College of Health Medicine, Kaya University, Gimhae 50830, Korea; choiye00@naver.com; 2 Department of Occupational Therapy, College of Health Medicine, Kaya University, Gimhae 50830, Korea

**Keywords:** early adolescents, sensory processing, leisure participation, correlations

## Abstract

Sensory processing may be associated with adolescents’ preferences for different leisure activities. However, knowledge about how different sensory processing patterns may relate to adolescents’ participation in leisure activities is scarce. This study sought to investigate the relationship between sensory processing and leisure participation in early adolescents. Study participants were typical early adolescents aged from 11 to 12 years (mean = 11.88 ± 0.33, *n* = 140). The Adolescent/Adult Sensory Profile (AASP) and Children’s Assessment for Participation and Enjoyment (CAPE) were used to determine the participants’ sensory processing abilities. Correlational and multiple regression methods were employed to analyze the relationship between sensory processing and leisure participation. There were significant positive relationships between sensory seeking and participation (r = 0.177–0.350, *p* = 0.000–0.037). There were also significant negative relationships between low registration, sensory sensitivity, and overall participation (r = −0.202, *p* = 0.017, r = −0.212, *p* = 0.012). We found that formal activities, skill-based activities, and self-improvement activities were the main distinguishing factors between sensory processing types. Results suggest that sensation seeking and sensory sensitivity from the AASP were predictive of leisure participation. This study provides evidence to inform practices regarding the association of sensory processing and leisure participation and supports the need for assessing sensory processing in early adolescents.

## 1. Introduction

Adolescence is one of the most important periods of human development. During this period, decisions that are made and actions taken can implicate on the rest of a person’s life. Although early adolescents—between 10 and 14 years of age—undergo some of the most significant and rapid physical, emotional, social, and cognitive changes in life, the needs of younger adolescents, who face greatest risks and complications related to unhealthy behaviors, are often overlooked. The literature has also failed to consider adolescent health behaviors along a life course that begins before a specific behavior is initiated [1]. Compared to studies focusing on adolescents aged 15 to 19, relatively fewer studies have been conducted for adolescents aged 10 to 14 [2].

Recent neurobiological research has shown that when children begin their pubertal transition, the brain experiences significant neural circuitry reorganization that impacts an individual’s processing of emotions, risks, rewards, and social relationships [3,4]. This is a dynamic period because of hormonal, pubertal, and social structure changes, as well as due to changes in relation to social structures. Although cognitive development increases during adolescence, neurophysiological development continues well into early adulthood. These neurophysiological developments occur in the brain’s prefrontal lobe, an area responsible for the “consciousness of the brain,” planning for future actions, considering consequences, and regulating emotional impulses. Additionally, this period is characterized by increased testosterone levels, which has a significant impact on the amygdaloid body that controls fear and aggression and has been associated with triggering anger, sexual interest, dominance, and territorial behavior [5]. The amygdaloid body analyzes sensory information such as sound, smell and image, and generates emotions based on the analyzed sensory stimuli. Unlike adults, adolescents experience difficulty in exercising self-regulation, leading to heightened impulses that may prove to be stifling, a strong instinctive power, unpredictable mood swings, confused emotions, and fear [5,6].

Emotions are responses of the nervous system to sensory information. They occur when sensory stimuli are interpreted and integrated through sensory processing [7]. Differences in the activities of the nervous system lead to differences in the methods of processing sensory information, which affects the mood, personality, and lifestyle of individuals in everyday life [8,9,10]. Multisensory binding in children and adolescents can affect their development. However, other sensory abnormalities can be more immediate and can include painful responses to everyday sensory stimuli, such as light and sound [11]. The development of the nervous system in adolescents is different to that of children and adults, and the discrepancies lead to differences in sensory processing or the degree of participation in daily activities such as leisure activities. Abnormal sensory responses are possible predictors of the severity of social disorders. A higher severity of sensory problems leads to a higher number of social problems. This means that sensory processing problems affect a child’s ability to participate in social and leisure activities [12]. Participating in leisure activities during adolescence is important for maintaining health and to maintain quality of life. Furthermore, it is associated with adolescents’ self-esteem, autonomy, and identity [13,14,15]. Adolescents tend to spend more than half of their waking hours in some form of leisure activity [16,17,18]. How can free time spent in early adolescence affect leisure activities in adulthood? Physically active children and adolescents have been reported to become active adults [19,20]. In addition, the degree of participation in leisure activities affects the risk of developing diseases. For example, while sufficient physical activity has been shown to contribute to positive physical and mental health [21], excessive media use has been associated with poor mental and physical health [22]. For this reason, it is important to investigate the leisure activities of healthy children and adolescents and to intervene early, if necessary.

Perceiving environmental stimuli or changes in the environment and adapting to these changes is a natural process in human life. These processes occur throughout an individual’s life and are not limited to a specific age [23]. Sensory processing and intervention and an evaluation of play or leisure have been conducted in children [24,25]. Watts, Stagnitti, and Brown [26] completed a systematic review investigating the relationship between sensory processing and play. This review showed that all the studies that were included had a concept of sensory processing, play, or both. Ismael, Lawson, and Cox [27] reported that children with different sensory processing patterns preferred both similar and distinct leisure activities. However, little is known about sensory processing or the relationship between sensory processing and play in early adolescents. Early adolescents have different brain motions to adults. Thus, it is predicted that the correlation between sensory processing characteristics and an early adolescent’s personality may form a different pattern than that of an adult. Therefore, this study aimed to provide basic data to determine the correlation between sensory processing ability and leisure participation in early adolescents and to predict the possibility of a positive sensory processing approach for early adolescents, which is a pertinent social issue.

## 2. Materials and Methods

### 2.1. Participants

The participants in this study were elementary school students aged from 11 to 12 years. This study included adolescents with typical development, without a disability or diagnosis. If parents reported in the questionnaire that their children had a diagnosis or disability or received occupational, physical, speech, or psychological therapy for any concerns, these adolescents were excluded from the study. The adolescents and their guardian, from whom we received consent after explaining the objective of the study and the assessment, participated in this study. Among the 244 students who provided the consent, data from 140 were analyzed, excluding data from 15 students who met the exclusion criteria and 89 with missing responses. The final sample included 69 boys and 71 girls aged 11 to 12 (mean age = 11.88 ± 0.33). G*Power was used for power analysis for correlations and multiple regression analyses [28].

### 2.2. Instruments

#### 2.2.1. Children’s Assessment of Participation and Enjoyment (CAPE)

CAPE [29] was used to examine the way in which they participated in different activity types. It is a measure designed to assess participation in recreational and leisure activities outside of mandated school hours, for children, with and without disabilities, from 6 to 21 years of age. It consists of 55 items classified by domains (15 formal activities and 40 informal activities), and by activity types (12 recreational, 13 physicals, 10 socials, 10 skill-based and 10 self-improvements). Each leisure activity has five dimensions of participation: (i) diversity (whether the activity was performed in the past 4 months); (ii) intensity (how often); (iii) with whom (iv) where; (v) enjoyment. Recreational activities include puzzles, card games, and crafts; physical activities include martial arts, bicycling, skateboarding, and in-line skating; social activities include attending a party, spending time with people and visiting people; skill-based activities include making food, and swimming; and self-improvement activities include writing letters, reading, and completing a chore.

It takes 30–45 min to complete the evaluation. Standard data are not provided. The instrument has been developed to show the current levels of participation, describe current patterns of participation, and record changes over time. The reliability of the Korean version of CAPE used in this study is between 0.928 and 0.9763 (alpha coefficient) [30].

#### 2.2.2. Adolescent/Adult Sensory Profile (AASP)

AASP is a self-report measure of sensory processing for people of 11 years of age or older. It consists of 60 items. Each item describes a behavior related to an everyday sensory experience that is scored on a 5-point Likert scale, indicating how frequently a person responds to a sensation. The scale ranges from 5 (you almost always respond) to 1 (you almost never respond). Items are split into four quadrants—low registration, sensation seeking, sensory sensitivity, and sensation avoiding. Each item is divided into six sections: taste/smell processing, movement processing, visual processing, touch processing, activity level, and auditory processing. The result of the self-report allows for intervention planning, which takes into consideration the individual’s particular preferences regarding what the individual wants or needs to do in his or her life. There are three age-group charts—adolescents (11:0–17:11 years), adults (18–64:11 years), and older adults (65 years and older). Each quadrant has its own score [24]. Reliability statistics are between 0.639 and 0.775 (alpha coefficients), and validity statistics are between 3.58 and 4.51 (standard errors of measurement) [24]. In this study, the Korean version of CAPE was used to evaluate the participants’ sensory processing. Reliability statistics are between 0.660 and 0.804 (alpha coefficients), and the discriminant validity by binary logistic regression is 71.6% (classification accuracy) [31].

### 2.3. Procedures

This study was after acquiring the approval of the Institutional Review Board (IRB 322) of Kaya University. We requested the recruitment of research participants from 15 elementary schools in one large city and one small city, and 13 classes were selected from three elementary schools by school officials. A questionnaire about general characteristics, including questions about disability or diagnosis, and a consent form were provided to the participants’ guardians, and duly signed consent forms were received. Informed consent was obtained from all the participants involved after explaining the objective of the study and the assessment. We distributed the survey questionnaires (AASP, CAPE) in person, and provided a sufficient amount of time to understand the content, and the explanation of the assessment. The questionnaire was only provided to students whose consent forms were received. Participants were asked to complete two self-report assessments without missing any of the items. It took approximately one hour to complete both assessments.

### 2.4. Data Analysis

The data were entered into IBM SPSS Statistics (Version 21; IBM Corp., Armonk, NY, USA) for analysis. Because the data did not meet the assumptions of normality, Spearman’s rank correlation was conducted. For the analysis, we measured the scores of the five scales (Diversity, Intensity, With Whom, Where, and Enjoyment) and the two domains of CAPE, and the quadrant scores for AASP. The diversity scale scores for the five activities and the two domains of CAPE were converted to a percentage of the maximum possible score by dividing the diversity scale raw score by the total possible scores for the activity types or domains, which were then multiplied by 100 [32]. This was done to ensure the scores were commensurate before a data analysis. A regression analysis was completed for which the quadrant scores for AASP were the independent variables and the Enjoyment scale scores were the dependent variables. We used descriptive statistics for the result mean scores for two assessments. The descriptive statistics for each form were then compared to derive the results.

## 3. Results

In Table 1, we present the descriptive statistics for the CAPE results in the form of mean and standard deviations. High scores on the Diversity, Intensity and Enjoyment scales indicate participation levels, the frequency of participation, and preferences. Most participants preferred informal activities, whereas social activities had the highest levels of participation, frequency of participation, and preference.

Spearman’s correlation test was used to analyze the correlations between the scores for the five activity types in CAPE, overall participation, and the four quadrants of the AASP. The results are shown below in Table 2.

The Diversity, Intensity and Enjoyment for overall participation, and for almost all of the five activity types of CAPE, showed a particularly positive correlation between the sensation seeking quadrant and skill-based activities. The Enjoyment scale showed a high correlation at a significance level of ≤ 0.001. An increase in the sensitivity seeking propensity was related to an increase in leisure activity participation rates. Low registration and sensory sensitivity were related to the decreased enjoyment scale of overall participation, specifically for skill-based and self-improvement activities (*p* < 0.001).

Sensory sensitivity was correlated with ‘with whom’ and ‘where’ (Table 3). Table 4 presents the results of the regression analysis. The quadrant scores of AASP were entered as independent variables, and the dependent variable was the CAPE Enjoyment score, which significantly correlated with a lower test score.

## 4. Discussion

CAPE is a comprehensive assessment tool composed of items that facilitate a familiar acceptance of play activities that can be used in clinical and research environments. It can be applied to children with or without physical disabilities and has low specificity for cultural differences [29]. We used the CAPE results to investigate the social participation patterns of early adolescents to identify useful information for intervention development. This study provides information about the nature of the relationship between the typically developing early adolescents and their leisure participation. Sensation seeking—one of the four sensory processing patterns—predicted an increased interest in leisure activity participation in early adolescents, while sensory sensitivity predicted a decreased interest. Sensation seeking was closely related to active participation or interest in most types of leisure activities. The study also indicated that leisure activities with low participant registration and sensational sensitivity tendencies were avoided. In particular, there was a remarkable difference in the sensory processing patterns during the engagement of the two domains—formal and informal activities—and sensory processing was related to ‘with whom’ and ‘where’ in leisure. This is clear evidence that early adolescents’ leisure participation is affected by their sensory processing ability. This provides preliminary information to parents and therapists about the potential leisure activity types that may be affected by sensory processing.

Play preferences appear to be influenced by sensory preferences [33,34]. Among the sensory processing patterns, sensation seeking was positively correlated with all the domains and activity types of leisure, except for the diversity and intensity scales. Lawson and Dunn [33] found significant differences between toy categories and the sensation seeking scores of participants. Sensory seekers are attracted to environments that create additional stimuli or provide sensory stimuli to meet neurological thresholds and consider sensory experiences enjoyable. Children who were sensory seeking sought toys that met their sensory needs, such as creative art toys or building blocks. They were easily bored in low-stimulation environments [24]. Therefore, sensation seeking is a personality trait that acts within brain physics and genetic programs that responds to individual history and environmental conditions [10]. It has been predicted that adolescents will participate in several leisure activities that reflect their preference with a more developed strategy of sensory regulation compared to children.

In this study, sensation seeking correlated with all types of activities but showed a relatively low correlation with social activities (talking on the phone, going to a party, etc.) among the five types of activities. Children with sensory processing issues present a level of delay in play, especially in the complexity of their social play and the reduction in time associated with toys and objects [33]. Social activities based on social competence and peer acceptance require skills such as identifying the needs of the social environment (e.g., appropriate language and non-verbal behavior), participating in actions responding to these needs, recognizing other people’s reactions, and adjusting their feedback to future environments [35]. Children with sensory seeking issues sought out play materials that could be interpreted as having sensory properties [33]. Sensory-seeking adolescents are thought to focus on their own sensory needs and are less sensitive to changes in the environment, including people and feedback. Thus, they have fewer positive experiences in social activities than other activities, leading to a lower preference for social activities.

The present study results confirmed that adolescents with a propensity towards low registration and sensory sensitivity showed a low preference for overall leisure participation. Among the five activities, they had low preferences for skill-based and self-improvement activities. Skill-based activities include activities that involve learning particular skills from others, such as dancing, participating in community organizations, and singing, or activities that provide various sensory stimuli. Adolescents with a low registration tend to miss responses to high-intensity or low-intensity stimuli and may not enjoy activities that require quick responses to stimuli. Meanwhile, people with sensory sensitivity easily experience distractions and discomfort caused by stimulation [24]. Therefore, they do not prefer to participate in skill-based activities as they require excessive attention to environmental stimuli such as music or noise or information from others who participate in an activity.

Self-improvement activities are related to schoolwork, including paperwork-based activities, such as doing homework, getting extra help from a tutor, and writing a story. People with low registration are relatively flexible and comfortable in an environment with multiple sensory stimuli [24] but may omit instructions or information received from others during schoolwork or assignments. A high level of environmental awareness and the ability to distinguish or pay attention to details are the key advantages of sensory sensitivity [24]. However, this may interfere with concentration, making it difficult to perform tasks consistently.

Ismael, Lawson, and Cox [32] studied children aged 6–14 years and reported results that were similar to the findings of this study. Low registration quadrant scores were negatively correlated with CAPE’s overall diversity scores, and sensory sensitivity quadrant scores were negatively correlated with a preference for social and skill-based activities. However, there was no correlation between low registration and any specific type of activity, and the correlation between sensory sensitivity and social activities was insignificant. Ismael, Lawson, and Cox [32] focused on diversity in their analysis. The study participants included young children and children with different sensory processing patterns who received individualized educational programs (IEP) and occupational therapy services. Young children and children receiving IEP may differ from early adolescents, in areas such as leisure preferences, skills, and participation abilities [32].

The sensory processing pattern also affected domain preference. As described above, adolescents with sensory sensitivity and low registration showed a lower preference for overall participation. However, they showed even lower preferences for formal leisure activities. More sensitive adolescents do not prefer skill-based activities because they have a high tolerance for long participation periods and enjoy activities with high sensory demands, such as dancing and swimming. They also care about their previous experience in such activities and may experience fear of failure, sensory stimuli, and other environmental demands [32]. Adolescents with low registration indicate that they do not enjoy or participate in formal leisure activities as they do not easily notice sensory demands from others or their environments [24].

For this reason, adolescents with sensory sensitivity, low registration, and sensation avoiding patterns showed a negative correlation for the ‘With whom’ and ‘Where’ scales. The results indicated that adolescents who are sensory sensitive or do not recognize sensory stimuli often prefer to enjoy leisure at home.

Adolescents who are more sensory sensitive prefer leisure activities that they can do by themselves at home. Adolescents develop independent self-regulation strategies against the discomfort they experience in the environment and device-coping mechanisms, including mental preparation (such as anticipation and confrontation) and cognitive strategies (such as avoidance, choice of activity, or organization of the environment) [36]. As adolescents with a high sensory sensitivity tend to detect and respond to stimuli more so than most people, they demonstrate increased efforts to participate in specific situations [37] and pre-set the environmental conditions of leisure activities as a management strategy for unpredictable sensory inputs.

Sensory processing patterns may affect the decision to participate, the frequency of participation, and an interest in specific leisure activities. Adolescents with these sensory issues may experience an imbalance in leisure participation compared to adolescents with typical development. In a systematic review [26] that investigated the relationship between sensory processing and play in children, there was a correlation between sensory processing skills and play skills. The main factors in play were poor attention, sensory seeking behaviors, caution during tasks, and limited play repertoire. However, there have been limited studies on the relationship between leisure and sensory processing in adolescents, especially for early adolescents.

The following are significant and novel findings of this study that distinguish it from existing research on children and adolescents:

1. There was a significant correlation between sensory processing and self-improvement activities, including learning and paperwork-based activities. Adolescents with concentration issues, that is, those who tend to omit information regarding school assignments and documents and instructions from others, or those who are easily distracted by visual or auditory stimuli, may have reduced learning achievements or reduced motivation to participate. This may lead to difficulties in schoolwork, which is the primary occupation of early adolescents.
2. Previous studies with children [27] showed no correlation of the “With whom” and “Where” scales with sensory processing patterns. However, this study showed patterns in their environmental preference for leisure activities, which was just as important as the type of leisure activities. This is because activities performed at home are predictable, and the surrounding sensory stimuli are familiar, which reduces adolescents’ cognitive and emotional load and minimizes negative behavior. Thus, it is the preferred leisure setting for adolescents equipped with self-regulation strategies through experience [38]. This finding indicates that it is necessary to consider the sensory environment to promote diverse leisure activities among adolescents.
3. The enjoyment scale of CAPE had the highest correlation with the sensory processing of adolescents in this study. As the participants of this study were school-age adolescents, there was no significant correlation of sensory processing with diversity and intensity scales (response to the leisure that they are participating in at the moment) that are affected by physical and time constraints. Enjoyment can be viewed as a predictor of pattern in individuals who find it difficult to participate in leisure activities according to their personal preferences due to schoolwork or other occupations.

The relationship between sensory processing and play has been reported to be neither simple nor clear [39]. An individual’s participation in play and leisure activities is influenced by personal and environmental factors and individual health conditions [40,41]. Although this study shares the views of previous studies, therapists working with adolescents need to understand sensory processing and the effect of sensory processing on participation. There is a relationship between the human nervous system and self-regulation strategies, and the interactions between these functions yield four basic patterns of sensory processing. Sensory processing patterns are unique characteristics of each person and not a diseases or conditions that require treatment [42,43]. Understanding an individual’s sensory processing characteristics is essential to promoting their participation in important activities related to their families, schools, and communities. This is true for adolescents with sensory processing disorders or extreme sensory differences and for typical adolescents participating in leisure activities.

This study investigated the sensory processing characteristics and their impact on leisure participation during a specific period of life. The study was performed with early adolescents in a small age range. The small age range of the participants may have served as both a strength and weakness of this study. The findings of this study do not provide an extensive understanding of sensory processing, leisure preference, and participation in adolescents across the age range. However, as the present study focused on early adolescents, it provides information for understanding the characteristics specific to their age range. As CAPE and AASP are self-report assessments conducted simultaneously, they may have affected the concentration and fatigue level of the participants. Further studies are necessary, exploring sensory processing and the participation of adolescents with sensory processing disorders or extreme sensory differences, as they are the main targets for assessment and intervention services in clinical practice.

## 5. Conclusions

The sensory preferences of early adolescents are associated with leisure enjoyment. Sensory seeking individuals showed a preference towards leisure participation, as compared to those with sensory sensitivity or low registration. To be more specific, they did not prefer skill-based self-improvement, and formal activities related to learning or situations where the external environment of others provided sensory stimuli. In addition, sensitive adolescents preferred leisure activities in an environment with less exposure to specific sensory stimuli. This means that sensory processing can affect the tasks and experiences of adolescents. Experts need to consider the characteristics of an individual’s mode of sensory processing and the areas they focus on with regard to adolescents.

## Figures and Tables

**Table 1:** Descriptive statistics for the CAPE Scores.

	Overall	Domain		Activity Type				
		Formal	Informal	Recreational	Physical	Social	Skill-Based	Self-Improvement
Diversity	23.0 ± 69.95	5.59 ± 3.37	17.47 ± 7.31	47.08 ± 20.43	34.06 ± 21.93	53.21 ± 24.93	36.07 ± 24.48	42.00 ± 24.96
Intensity	2.00 ± 0.86	1.81 ± 1.14	2.08 ± 0.87	2.27 ± 1.04	1.64 ± 1.14	2.43 ± 1.21	1.72 ± 1.27	2.04 ± 1.21
Enjoyment	1.88 ± 0.34	1.78 ± 0.41	1.89 ± 0.33	16.17 ± 3.96	1.94 ± 0.47	2.02 ± 0.44	1.84 ± 0.49	1.61 ± 0.44
With whom	2.66 ± 0.59	-	-	-	-	-	-	-
Where	2.47 ± 0.60	-	-	-	-	-	-	-

**Table 2:** Correlation matrix between AASP scores and CAPE.

CAPE			Low Registration	Sensation Seeking	Sensory Sensitivity	Sensation Avoiding
Overall participation		Diversity	−0.046	0.262 **	0.016	0.157
		Intensity	−0.046	0.302 ***	0.045	0.148
		Enjoyment	−0.202 *	0.350 ***	−0.212 *	−0.089
Domain	Formal	Diversity	−0.020	0.271 **	0.047	0.153
		Intensity	−0.062	0.249 **	0.013	0.157
		Enjoyment	−0.225 **	0.331 ***	−0.243 **	−0.089
	Informal	Diversity	−0.086	0.209 *	−0.047	0.135
		Intensity	−0.021	0.298 ***	0.081	0.150
		Enjoyment	−0.186 *	0.305 ***	−0.188 *	−0.003
Activity types	Recreational	Diversity	0.010	0.155	0.065	0.093
		Intensity	0.015	0.150	0.116	0.112
		Enjoyment	−0.138	0.271 ***	−0.157	−0.013
	Physical	Diversity	−0.075	0.204 *	−0.002	0.165
		Intensity	−0.060	0.180 *	0.037	0.169 *
		Enjoyment	−0.138	0.272 ***	−0.155	−0.011
	Social	Diversity	−0.028	0.247 **	0.010	0.146
		Intensity	−0.052	0.255 **	0.005	0.095
		Enjoyment	−0.134	0.260 **	−0.145	0.026
	Skill-based	Diversity	−0.034	0.221 **	−0.034	0.153
		Intensity	−0.034	0.248 **	−0.005	0.075
		Enjoyment	−0.170 *	0.323 ***	−0.226 **	−0.063
	Self-improvement	Diversity	−0.045	0.177 *	0.020	0.047
		Intensity	−0.031	0.248 ***	0.161	0.089
		Enjoyment	−0.225 **	0.267 ***	−0.181 *	0.014

* *p* <0.05, ** *p* < 0.01, *** *p* < 0.001.

**Table 3:** Correlation matrix between AASP Scores and CAPE With whom and Where.

AASP	With Whom	Where
Low registration	−0.099	−0.192 *
Sensation seeking	0.033	0.001
Sensory sensitivity	−0.169 *	−0.281 ***
Sensation avoiding	−0.086	−0.187 *

* *p* < 0.05, *** *p* < 0.001.

**Table 4:** Regression analysis results for AASP scores as predictors of Enjoyment.

	Enjoyment			
	B	SE	Beta	*p*
Low Registration	−0.009	0.128	−0.189	0.082
Sensation seeking	0.019	0.005	0.434	0.000
Sensory Sensitivity	−0.013	0.003	−0.298	0.019
Sensation Avoiding	0.006	0.005	0.139	0.185

*R*^2^ = 0.246, F = 10.995, *p* = 0.000, Durbin-Watson = 2.095.

## Data Availability

The data presented this study will be provided on request.
